# Extensive acute cutaneous graft versus host disease: A rare case report of survival

**DOI:** 10.1002/ccr3.7545

**Published:** 2023-06-13

**Authors:** Prajwal Pudasaini, Sushil Paudel, G. C. Sagar, Sadiksha Adhikari, Neeraj Thapa, Bibechan Thapa

**Affiliations:** ^1^ Department of Dermatology Civil Service Hospital Kathmandu Nepal; ^2^ Nepal Medical College Teaching Hospital Kathmandu Nepal; ^3^ Department of Emergency and Inpatient Care Nepal National Hospital Kathmandu Nepal

**Keywords:** cutaneous graft versus host disease, hematopoietic stem cell transplant

## Abstract

Graft versus host disease (GVHD) is an immunologically mediated condition seen in allogeneic hematopoietic stem cell transplant (HSCT) recipients. Because of the rarity of the disease, nonspecific presentation, and lack of clinicopathological correlation, its diagnosis is often delayed and prompt treatment is deferred, with increased mortality.

## Graphical abstract

Extensive acute cutaneous GVHD.

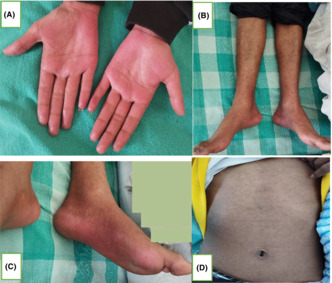

## 1 BACKGROUND

Graft versus host disease (GVHD) is an immunologically mediated polymorphically presenting condition manifesting with widespread systemic, cutaneous, and mucosal involvement.^1^ It occurs mostly in allogeneic hematopoietic stem cell transplant (HSCT) recipients, ascribable to cell‐mediated programmed destruction by cytotoxic T lymphocytes.^2^ GVHD is etiologically linked to a minor mismatch in the human leukocyte antigen (HLA) locus. Regarding the onset and incidence of GVHD, factors such as old age, donor tissue from HLA unmatched individuals lead to increased occurence and severity of acute cuteaneous GVHD, T‐cell replete versus deplete, or type of conditioning procedure such as reduced intensity radiotherapy or chemotherapy also aids in its causation.^3^ As around 20,000 allogeneic HSCTs are performed annually, with 66 transplants conducted in a single government‐based tertiary center in Nepal, clinicians need to be vigilant about the varied clinical features of GVHD.^4^, ^5^ Multiple organs are involved in GVHD such as the liver, gastrointestinal tract, and skin, in which the latter is almost always involved. Skin involvement can also be varied based on acute GVHD (aGVHD) or chronic GVHD. In which case, aGVHD presents mostly with maculopapular exanthem with predominant involvement of extremities and trunk, and in extreme forms with toxic epidermal necrolysis (TEN) like skin denudation linked to high mortality.^2^, ^6^, ^7^

GVHD, due to its varied presentation is difficult for clinicians to diagnose, which more often than not, leads to fatal consequences.^7^ This case report reiterates the importance of clinical acumen in preventing nonrelapse associated mortality in hematological cancer patients post allogeneic HSCT in resource‐poor settings.^8^ Herein, we report a case of aGVHD with extensive skin exfoliation, penile ulcer, and survival attributed to good clinical acumen, histopathological correlation, and prompt treatment.

## 2 OBSERVATION

**FIGURE 1:**
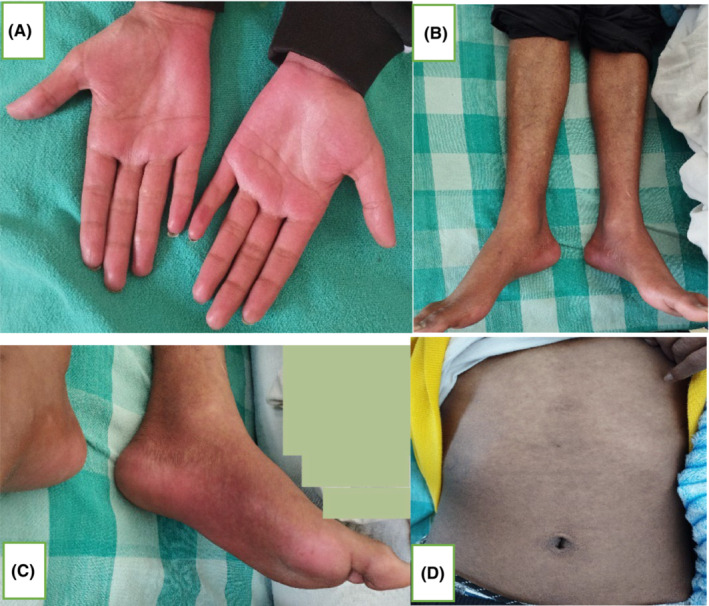
(A–D) Erythematous maculopapular exanthem involving palmoplantar region, extremities, and trunk.

**FIGURE 2:**
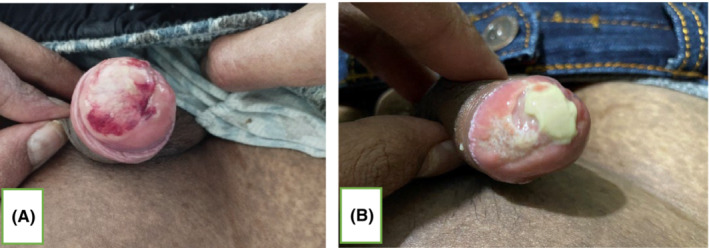
Solitary ulcer with whitish slough over the glans penis.

**FIGURE 3:**
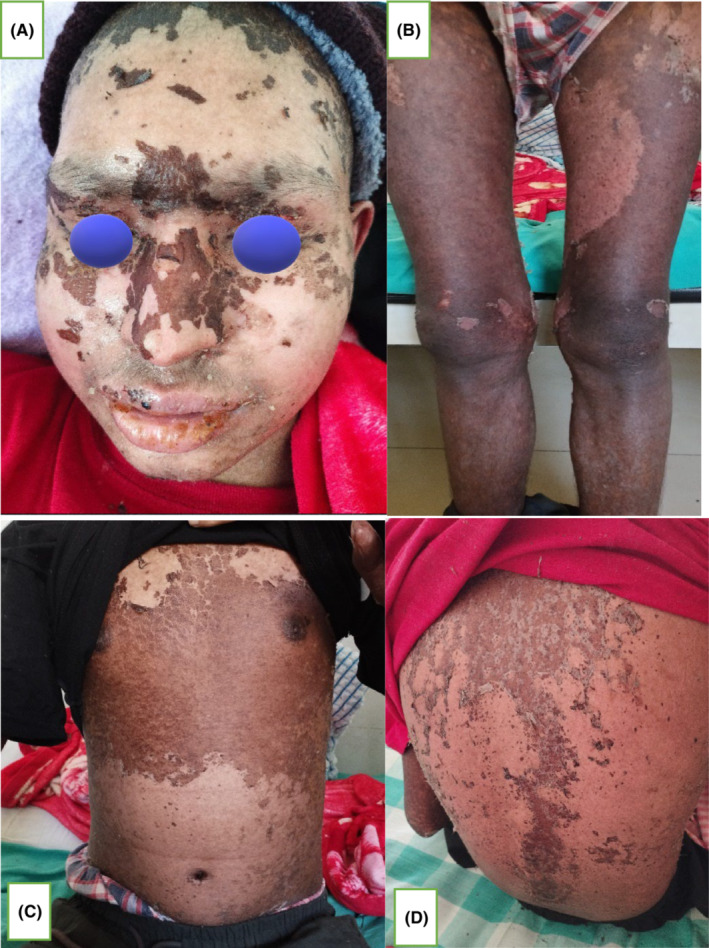
(A–D) Post inflammatory desquamation over the face, trunk, and extremities post 10th day of the disease.

An 18‐year‐old male, known case of acute myeloid leukemia (AML), post 58th day of allogeneic HSCT (matched related donor: father, HLA match: 5/10), presented with mildly pruritic, reddish lesions over the bilateral palmoplantar region, with gradual progression of lesion to involve forearm, legs, trunk, face, scalp, and ears over a period of 10 days (Figure 1A–D). Initially, multiple pinhead sized, flat lesion with red color was noted over the bilateral palm that became confluent to involve and extend to 95% of body surface area (BSA). Also, a painful solitary ulcer with whitish slough over the glans penis was present (Figure 2A,B). There was associated swelling of face, lips, and limbs. Lesions further progressed over a period of 10 days with desquamation of skin color to brownish scales over the lesion site and extension of penile erosion (Figure 3A–D) There was no history of vomiting, diarrhea, abdominal pain, or yellowish discoloration of the body. On examination, erythematous maculopapular exanthem was noted over generalized body with widespread skin denudation, largest plaque of 10 * 5 cm in size irregular shaped over back in the mid‐vertebral line of the spine on genital examination irregular erosion of 1 * 0.5 cm in diameter was noted over the glans penis with whitish thick slough. Laboratory investigations showed pancytopenia, hyponatremia, and slightly increased alkaline phosphatase level = 167 U/L (range 30–120) suggestive of mild cholestasis. Biopsy showed vacuolar change of basal layer of cells, hydropic changes, keratinocyte apoptosis, and band‐like lichenoid dermal lymphohistiocytic infiltrates.

**FIGURE 4:**
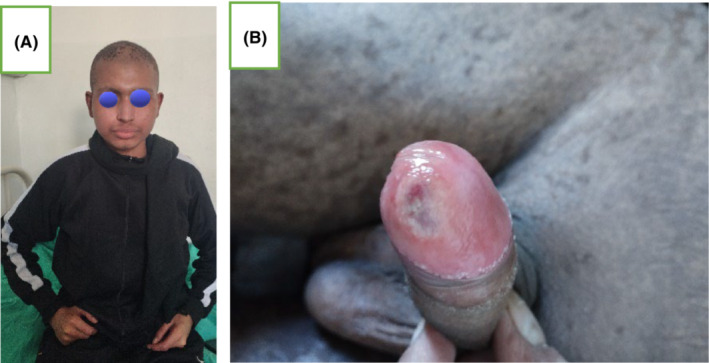
(A) Complete resolution of skin lesion; (B) healed penile erosion with post‐inflammatory mucosal hyperpigmentation.

Patient was treated with oral methylprednisolone 5 mg twice daily tapered over 3 weeks and maintained with Tacrolimus 1 mg twice daily for 6 months, along with cotrimoxazole, valganciclovir, ursodeoxycholic acid for cholestasis, and sodium chloride tablet for hyponatremia. Topical mometasone furoate ointment was locally applied over erosion of the glans penis twice daily for 2 weeks with healing of lesion post treatment. The involved cutaneous and penile erosion healed over 3 weeks with normal underlying skin devoid of any pigmentation or scarring (Figure 4). New lesions of aGVHD have not evolved for 6 months until now and patient is under remission for AML.

## 3 DISCUSSION

GVHD is an immunologically mediated condition with varied systemic, cutaneous, and mucosal clinical features. It occurs mostly in allogeneic HSCT recipients, due to T‐cell‐mediated cytotoxicity.^1^, ^2^ The cause of this T‐cell‐mediated cytotoxicity is largely attributed to minor mismatch in the HLA locus.^3^ As in our case, HLA match of 5/10 could be attributed to the occurrence of aGHVD. Pathogenically, its occurrence is linked to the activation of an antigen presenting cell (APC), proliferation of T lymphocytes and cytotoxic destruction of organs such as the liver, gastrointestinal tract, and skin. In addition, host factor such as age or donor factors such as related versus unrelated, T‐cell replete versus deplete, or type of conditioning procedure such as myeloablative radiotherapy or chemotherapy also aid in its causation.^7^, ^9^, ^10^ As in our case, GVHD can occur even in matched related donors, despite the use of filgrastim in chemo myeloablative conditioning regimen, possibly attributable to minor HLA mismatch.^11^ As in our case, aGVHD present as maculopapular exanthem with erythroderma involving almost 95% BSA and penile ulcer. Usually, extensive erythrodermic patients with systemic involvement have high mortality.^1^, ^6^, ^12^ However, on the contrary, our patient with extensive skin denudation, mucosal involvement with cholestatic change survived fatality with no systemic consequences or relapse till date. As in our case, lesions are initially acral followed by involvement of face, scalp, ear, and trunk, which can be mildly pruritic. This telltale sign of acral initiation and subsequent retrograde progression could be utilized for early diagnosis of the disease.^1^, ^12^ Post‐inflammatory desquamation with extensive skin denudation and erythroderma is an inevitable sequela in severe forms, linearly correlated to mortality.^12^ Also, mucosal involvement presenting as oral aphthous ulcer or penile erosion and slough are a frequent manifestation in aGVHD.^13^ Systemic features such as diarrhea, vomiting, abdominal pain, and jaundice usually co‐occur concurrently with skin manifestation, which was absent in our case.^2^, ^6^, ^7^ Laboratory investigations usually show hyperbilirubinemia and transaminitis.^1^ In our patient, alkaline phosphatase level was slightly increased which depicted a feature of cholestasis. Biopsy usually shows keratinocyte apoptosis, vacuolar degeneration of basal layer cells, hydropic changes, and band‐like lichenoid dermal lymphohistiocytic infiltrates as in the histopathological section of our case.(Figure 2)^2^ However, diagnosis will often rely on corroboration of clinical corroboration and clinical features, as histopathological mimics of aGVHD are often confounding.^14^

aGVHD is treated with oral or intravenous corticosteroid, in dose of 1 mg/kg twice daily, in tapering dose, along with prophylactic maintenance with calcineurin inhibitor such as tacrolimus or cyclosporine. Corticosteroid‐resistant cases can be alternatively treated with immunosuppressant such as mycophenolate mofetil, tumor necrosis factor alpha inhibitors, Janus kinase inhibitor (JAK inhibitor) such as ruxolitinib or commercially available mesenchymal stem cell product.^3^, ^15^ Most often the severe sequelae are mitigated with timely diagnosis and treatment. And in about half of the cases control of the disease is fortuitously achieved. Topical steroid ointment can be applied locally for limited cutaneous or mucosal lesion with healing of lesion post treatment without pigmentary or scarring sequelae as in our case.

GVHD, due to its varied presentation, is difficult for clinicians to diagnose, which can lead to unforeseen complications including mortality.^12^ This case report reiterates the importance of clinical acumen and clinicopathological correlation in preventing non‐relapse associated mortality in hematological cancer patients who have underwent allogeneic HSCT in resource‐poor settings.^8^ Herein, we report a case of aGVHD with extensive skin exfoliation, penile ulcer and survival attributed to good clinical acumen, histopathological correlation and prompt treatment. This case report iterates the importance of technique in lieu of technology in resource‐poor tertiary center of low‐income countries like Nepal.

## 4 ETHICAL STATEMENT FOR CLINICAL CASE REPORTS

The authors assure that the following are fulfilled:

1. This material is the authors' own original work, which has not been previously published elsewhere.
2. The paper is not currently being considered for publication elsewhere.
3. The paper reflects the authors' own research and analysis in a truthful and complete manner.
4. The paper properly credits the meaningful contributions of coauthors and coresearchers.
5. The results are appropriately placed in the context of prior and existing research.
6. All sources used are properly disclosed (correct citation). Literally copying of text must be indicated as such by using quotation marks and giving proper reference.
7. All authors have been personally and actively involved in substantial work leading to the paper, and will take public responsibility for its content.

The violation of the Ethical Statement rules may result in severe consequences.

To verify originality, this article may be checked by the originality detection software.

The authors agree with the above statements and declare that this submission follows the policies of Clinical Case Reports as outlined in the Guide for authors and in the Ethical Statement.

Date: 5/9/2023.

Corresponding author's signature: *prazol*.

## 5 CONSENT STATEMENT FOR CLINICAL CASE REPORTS

The authors have confirmed that patient consent has been signed and collected in accordance with the journal's patient consent policy.

The authors agree with the above statements and declare that this submission follows the policies of Clinical Case Reports as outlined in the Guide for authors and in the Ethical Statement.

Date: 5/9/2023.

Corresponding author's signature: *prazol*.

## AUTHOR CONTRIBUTIONS

**Prajwal Pudasaini:** Conceptualization; supervision; visualization. **Sushil Paudel:** Conceptualization; project administration; resources; writing – original draft; writing – review and editing. **G.C Sagar:** Conceptualization; supervision. **Sadiksha Adhikari:** Conceptualization; visualization. **Neeraj Thapa:** Investigation. **Bibechan Thapa:** Conceptualization; visualization.

## FUNDING INFORMATION

None.

## CONFLICT OF INTEREST STATEMENT

None.

## ETHICS STATEMENT

The authors declare that this submission follows the policies of Clinical Case Reports as outlined in the Guide for authors and in the Ethical Statement.

## CONSENT

Written informed consent was obtained from the patient to publish this report in accordance with the journal's patient consent policy.

## Data Availability

The data that support the findings of this study are openly available in Clinical Case Reports.
